# Reconstruction of rabbit mandibular bone defects using carbonate apatite honeycomb blocks with an interconnected porous structure

**DOI:** 10.1007/s10856-022-06710-2

**Published:** 2022-12-31

**Authors:** Keiko Kudoh, Naoyuki Fukuda, Kazuya Akita, Takaharu Kudoh, Natsumi Takamaru, Naito Kurio, Koichiro Hayashi, Kunio Ishikawa, Youji Miyamoto

**Affiliations:** 1 Department of Oral Surgery, Institute of Biomedical Sciences, Tokushima University Graduate School, Tokushima, Japan; 2 Department of Biomaterials, Faculty of Dental Science, Kyushu University, Fukuoka, Japan

**Keywords:** Carbonate apatite, Bone substitute, Bone remodeling, Honeycomb blocks, Mandibular reconstruction

## Abstract

Carbonate apatite (CO_3_Ap) granules are useful as a bone substitute because they can be remodeled to new natural bone in a manner that conforms to the bone remodeling process. However, reconstructing large bone defects using CO_3_Ap granules is difficult because of their granular shape. Therefore, we fabricated CO_3_Ap honeycomb blocks (HCBs) with continuous unidirectional pores. We aimed to elucidate the tissue response and availability of CO_3_Ap HCBs in the reconstruction of rabbit mandibular bone defects after marginal mandibulectomy. The percentages of the remaining CO_3_Ap area and calcified bone area (newly formed bone) were estimated from the histological images. CO_3_Ap area was 49.1 ± 4.9%, 30.3 ± 3.5%, and 25.5 ± 8.8%, whereas newly formed bone area was 3.0 ± 0.6%, 24.3 ± 3.3%, and 34.7 ± 4.8% at 4, 8, and 12 weeks, respectively, after implantation. Thus, CO_3_Ap HCBs were gradually resorbed and replaced by new bone. The newly formed bone penetrated most of the pores in the CO_3_Ap HCBs at 12 weeks after implantation. By contrast, the granulation tissue scarcely invaded the CO_3_Ap HCBs. Some osteoclasts invaded the wall of CO_3_Ap HCBs, making resorption pits. Furthermore, many osteoblasts were found on the newly formed bone, indicating ongoing bone remodeling. Blood vessels were also formed inside most of the pores in the CO_3_Ap HCBs. These findings suggest that CO_3_Ap HCBs have good osteoconductivity and can be used for the reconstruction of large mandibular bone defects.

**Graphical Abstract:**

**Figure:**
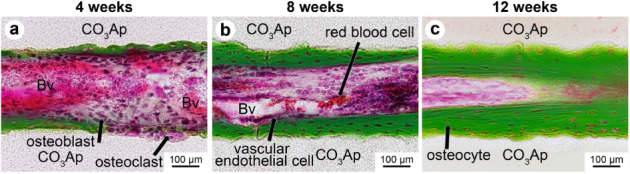
The CO_3_Ap HCB were gradually resorbed and replaced by newly formed bone.

## Introduction

The inorganic component of the human bone structure is not hydroxyapatite (HAp), but carbonate apatite (CO_3_Ap). We had, therefore, attempted to and succeeded in fabricating low-crystalline CO_3_Ap using a dissolution-precipitation reaction [1]. The CO_3_Ap fabricated by the reaction was replaced with bone in phase with bone remodeling [2, 3]. Bone remodeling denotes the replacement of old bone with new bone through sequential osteoclastic resorption and osteoblastic bone formation [4]. Unlike sintered hydroxyapatite (HAp), CO_3_Ap can be resorbed by osteoclasts [3, 5, 6]. Bone replacement of CO_3_Ap is thought to start from the resorption by osteoclasts in a manner similar to that of bone remodeling.

Kudoh et al. reported a first-in-human clinical trial of eight cases with one-stage sinus floor augmentation in which the CO_3_Ap granules, fabricated by the reaction, led to adequate new bone formation to support dental implants that were maintained for 1 year, with no implant failures or complications [7]. Nakagawa et al. first reported 13 cases of two-stage sinus floor augmentation in which the CO_3_Ap granules showed excellent biocompatibility and were replaced with bone in humans, as assessed via bone biopsy [8]. The survival and success rates of 17 implants after 3 years were 100%, with no corresponding complications [9]. Based on these clinical trials [7–9], the CO_3_Ap granules were approved as an artificial bone substitute and marketed by GC Corporation as Cytrans Granules^®^ in Japan and the United States. In Japan, no artificial bone substitutes had been previously approved for the reconstruction of bone defects adjacent to dental implants owing to their relatively poor osteoconductivity. CO_3_Ap granules have been approved in all dental and maxillofacial fields, including those adjacent to dental implants, owing to their higher osteoconductivity.

The composition and structure of bone substitutes are the key factors governing their osteoconductivity and replacement of bone ability. Moreover, porous bone substitutes are useful because they allow bone tissues and blood vessels to penetrate the pores, leading to higher osteoconductivity and quicker bone regeneration [10, 11]. Furthermore, a porous surface improves the mechanical integrated bond between the bone substitute and the surrounding natural bone, providing greater mechanical stability [12, 13]. Therefore, CO_3_Ap with porous structures is a promising bone substitute owing to its tendency to promote bone formation. Akita et al. succeeded in fabricating CO_3_Ap granules with porous structures using microfibers [14]. Additionally, Ishikawa et al. fabricated porous CO_3_Ap blocks with unidirectional continuous pores, called CO_3_Ap honeycomb blocks (CO_3_Ap HCBs) [15].

Bone defects in the oral and maxillofacial regions are caused by cancer resection, trauma, congenital malformations, progressive skeletal deformity, or orthognathic surgery. Bone reconstruction of specific regions is extremely difficult because of their complicated shapes. In the mandible, marginal or segmental mandibulectomy is performed for resecting odontogenic tumors (e.g., ameloblastoma), oral cancer, and mandibular osteomyelitis, including osteoradionecrosis and medication-related osteonecrosis of the jaw. Titanium reconstruction plates or autogenous bone grafts (e.g., from the fibula, scapula, iliac crest, or radial forearm osteocutaneous flap) are commonly used for the reconstruction of mandibular defects. Because titanium reconstruction plates are associated with risks such as infection and exposure of the plate, screw loosening, and plate fracture, they are used for temporary mandibular reconstruction. Autogenous bone grafts are the gold standard for the reconstruction of mandibular defects, which can be vascularized, such as in the fibula flap, or non-vascularized, such as in the anterior and posterior iliac crest grafts [16]. Autogenous bone grafts demonstrate potential for contouring and immediate dental implant placement and exhibit bone regenerative ability. However, this technique has some disadvantages, such as the need for a healthy donor site surgery, long surgical time, high costs, complications, and long hospital stay. Therefore, the reconstruction of mandibular defects using an artificial bone substitute that is gradually replaced by natural bone is desirable. As mentioned above, CO_3_Ap has excellent osteoconductive properties, biocompatibility, and the ability to be replaced with bone. In addition, the structure of CO_3_Ap HCBs promotes bone penetration along the unidirectional continuous pores from both lateral ends. In other words, CO_3_Ap HCBs might enable the reconstruction of large mandibular bone defects without autogenous bone grafting.

In this study, the tissue response and usefulness of CO_3_Ap HCBs were evaluated for the reconstruction of rabbit mandibular bone defects caused by marginal mandibulectomy.

## Materials and methods

### Materials

Calcium sulfate (CaSO_4_) powder (purity >97%) was purchased from Nacalai Tesque (Kyoto, Japan) and heated to 700 °C for 6 h before use. The wax-based binder (catalog number: K18) was purchased from Nagamine Manufacturing Co., Ltd. (Manno-town, Japan).

### Fabrication of CO_3_Ap HCBs

CO_3_Ap HCBs were prepared as previously described [17]. Briefly, CaSO_4_ powder was mixed with a wax-based binder at 150 °C for 3 h using a Labo Plastomill M roller mixer (Toyo Seiki Co., Nagano, Japan) fitted with a uniaxial extruder. The mixture was extruded at 95 °C through a honeycomb (HC) extrusion die with 150 μm windows and a 300 μm pitch to generate HC rods. The HC rods were each cut to 2 cm in length to fabricate the blocks. The HCBs consisting of the binder containing CaSO_4_ were heated to 900 °C in a box furnace and maintained for 24 h (Nitto Kagaku, Nagoya, Japan) to remove the binder. The CaSO_4_-containing HCBs were immersed in a mixture of 2 M NaHCO_3_ and 2 M Na_2_CO_3_ and stored in a VTEC-18 incubator (Isuzu CAP, Niigata, Japan) at 40 °C for 4 days for conversion to CaCO_3_. The CaCO_3_ HCBs were washed multiple times with water, immersed in a 1 M Na_2_HPO_4_ solution, and stored in an incubator at 80 °C for 7 days for conversion to CO_3_Ap. The CO_3_Ap HCBs were subsequently rinsed with water several times.

### Characterization of CO_3_Ap HCBs

The CO_3_Ap HCBs composition was determined using powder X-ray diffraction (XRD) analysis. Each sample was pulverized, and the XRD pattern was recorded using a D8 Advance diffractometer (Bruker AXS GmbH, Karlsruhe, Germany) with Cu Kα radiation at 40 kV and 40 mA. The sample was scanned in continuous mode over a diffraction range of 20°–60° (2θ).

The Fourier transform infrared (FT-IR) spectra were measured using a spectrometer (FT/IR-6200; JASCO, Tokyo, Japan) and the KBr disk method. The HCB structure was confirmed using stereomicroscopy and scanning electron microscopy (SEM) with S3400 N (Hitachi High Technologies, Tokyo, Japan). The acceleration voltage was set to 15 kV. Before analysis, the samples were coated with Au–Pd using an MSP-1S magnetron sputtering source (Vacuum Device Co., Ibaraki, Japan).

The carbonate content of the samples was measured using elemental analysis or a CHN coder (MT-6; Yanaco Analytical Instruments, Kyoto, Japan).

### Application of the CO_3_Ap HCBs for reconstruction of the rabbit mandibular bone defects

All animal experiments conformed to the guidelines of the Animal Ethics Committee of the Institute for Frontier Medical Sciences, Tokushima University. Experimental protocols were reviewed and approved by the Tokushima University Animal Studies Committee (approval number: T30-46). Nine male Japanese white rabbits (body weight: 3.0–3.5 kg; SLC, Shizuoka, Japan) were used in this study. The rabbits were subjected to general anesthesia via intravenous injection of ketamine (10 mg kg^–1^) and xylazine (3 mg kg^–1^).

**Fig. 1:**
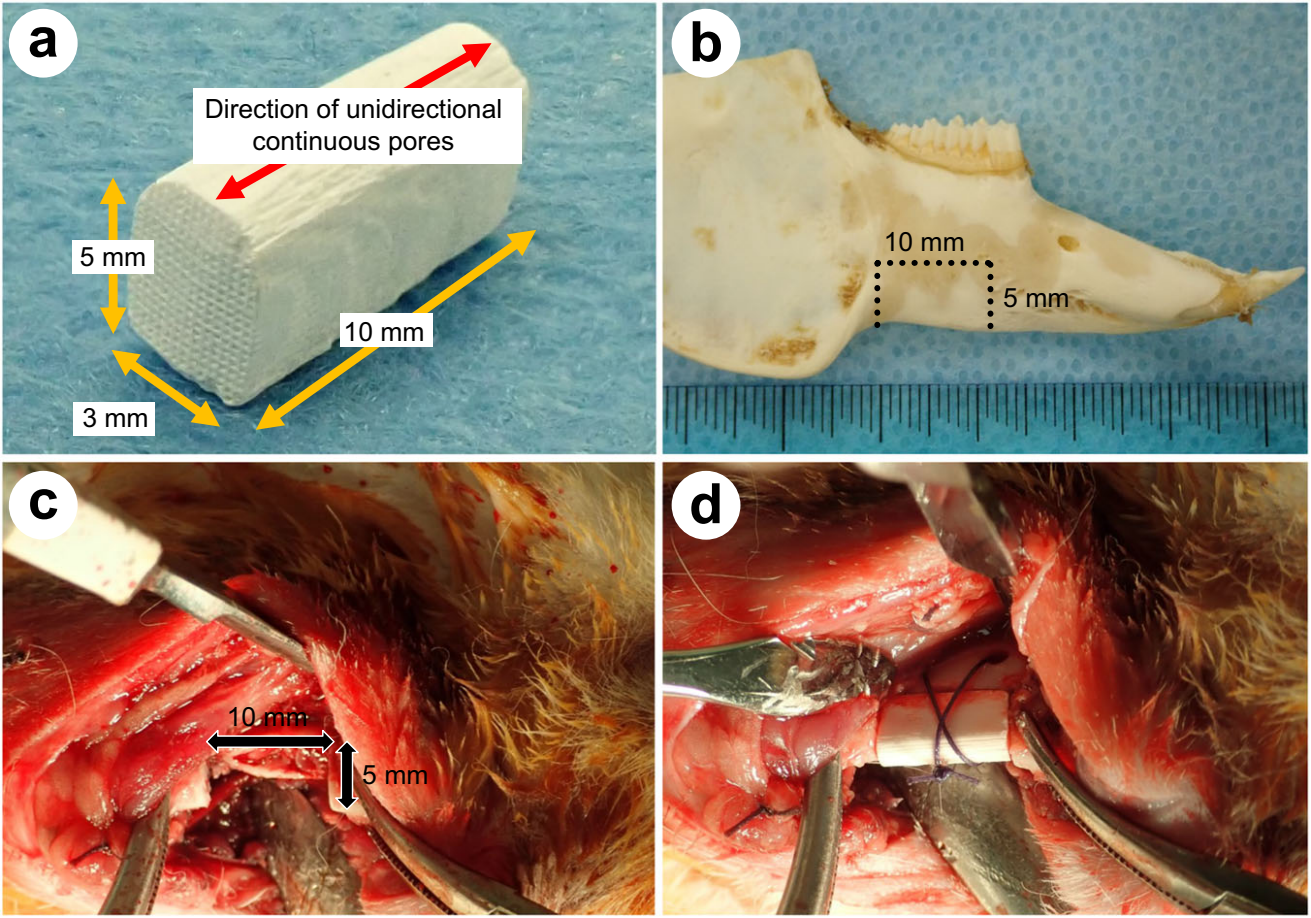
Application of the CO_3_Ap HCBs for reconstruction of the rabbit mandibular bone defects. CO_3_Ap HCB molded to a length, height, and width of 10, 5, and 3 mm, respectively (**a**). Excision image line of the rabbit mandibular defect (**b**). Inferior border osteotomy of the rabbit mandible (10 × 5 mm) (**c**). Grafting of the CO_3_Ap HCBs to the bone defect (**d**)

The skin of the buccal and lower sides of the mandible was shaved to remove the fur and was disinfected with a 10% povidone-iodine solution. After exposure of the mandibular body and ramus via the submandibular incision, 10 mm-long and 5 mm-deep bone defects were created using AESCULAP^®^ ELAN EC (B. Braun Melsungen AG, Hessen, Germany) and a reciprocating saw. Each CO_3_Ap HCB with a length of 10 mm, height of 5 mm, and width of 3 mm was implanted in each bone defect and fixed with surgical sutures (Fig. 1). The fasciae were then sutured. Inferior border osteotomy was performed bilaterally in each animal. Implantation of the HCB or an empty defect without an HCB was randomly performed. The rabbits were allowed unrestrained movement in their individual cages after recovery from anesthesia and a normal diet was provided.

### Microcomputed tomography (μ-CT)

The animals were euthanized via intravenous injection of pentobarbital (120 mg kg^–1^), and the mandibular bone was harvested at 4, 8, and 12 weeks after the operation. The excised mandibular bone was scanned using μ-CT with SkyScan1176 (Bruker microCT, Kontich, Belgium) at a source voltage and current of 50 kV and 500 μA, respectively. The image data were recreated using the NRecon Reconstruction software (Bruker microCT). Bone formation in the defects, including the specimens, was observed.

### Histological evaluation

Three samples from each group were fixed in 10% buffer formaldehyde for 7 days and dehydrated by immersion in graded ethanol solutions (from 70% to 100%). Finally, the samples were embedded in methyl methacrylate (WAKO Pure Chemical Corporation, Osaka, Japan). Sections were prepared for pathological analysis using a modified interlocked diamond saw (Exakt, Hamburg, Germany), and Villanueva Goldner stain was applied to all sections. All specimens were observed using an all-in-one microscope (BZ-X700; KEYENCE, Osaka, Japan).

**Fig. 2:**
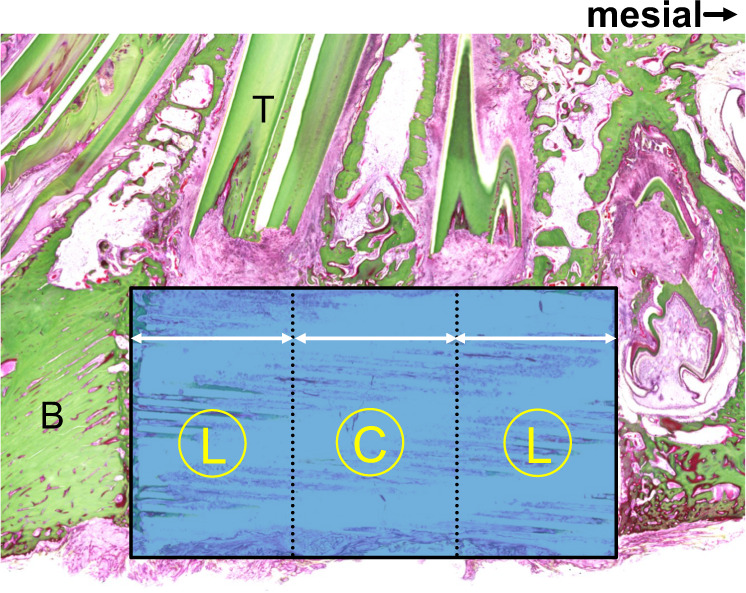
Compartments for the microscopic analysis of regenerated bone and residual CO_3_Ap in the defects. The right direction of the figure shows the mesial aspect. Specimens were divided into three parts, that is, the central part (C) and the lateral parts at both ends (L). “B” indicates the cortical bone of the inferior border of the mandible. “T” indicates tooth

To clarify the elongation of newly formed bone from the lateral parts at both ends of the bone defect, the defect area was divided into three parts: the central part and the lateral parts at both ends (Fig. 2). The percentage of newly formed bone (calcified bone stained in green) area in the defect was calculated using the ImageJ software (version 1.53a, Wayne Rasband, National Institutes of Health, USA). Furthermore, the percentage of the remaining CO_3_Ap area was calculated using the same method.

### Statistical analysis

For statistical analysis, one-way factorial analysis of variance and Fisher’s least significant difference *post-hoc* tests were performed using Kaleida Graph (version 4.1, Hulinks, Tokyo, Japan). Values are expressed as mean ± SD. Statistical significance was set at *p* < 0.05.

## Results

### Compositional and morphologic analysis

**Fig. 3:**
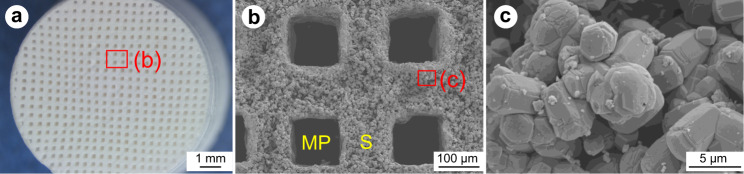
Stereomicroscopy and SEM images of CO_3_Ap HCBs. Stereomicroscopy image of a CO_3_Ap HCB (**a**). SEM image of a CO_3_Ap HCB (**b**, **c**). “S” and “MP” indicate a honeycomb strut and macropore, respectively. Magnified SEM images of the struts of the CO_3_Ap HCB (**c**)

Figure 3 shows stereomicroscopy (Fig. 3a) and SEM (Fig. 3b, c) images of CO_3_Ap HCBs. The CO_3_Ap HCBs exhibited a typical HC structure. In other words, the HCBs comprised square through-holes extending in one direction. The length of the macropore square side was 129 ± 6 μm, and the thickness of the strut was 130 ± 5 μm (Fig. 3b). A high-magnification image showed that the CO_3_Ap HCBs consisted of small crystallites that were mutually interlocked (Fig. 3c).

**Fig. 4:**
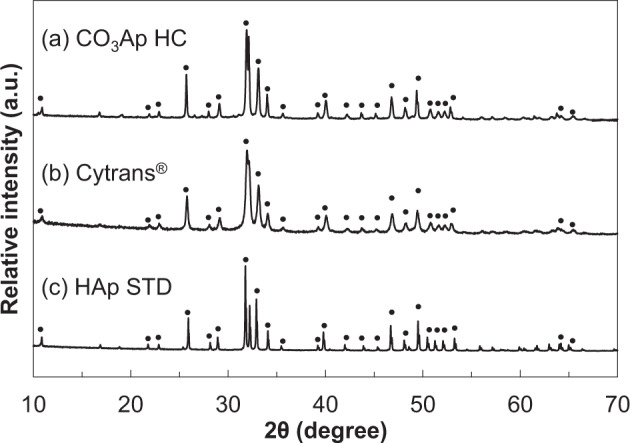
XRD patterns. CO_3_Ap HCBs (HC) (**a**), Cytrans Granules^®^ (Cytrans^®^; CO_3_Ap granules) (**b**), and HAp standard substance (STD) (**c**). Circles indicate the apatite diffraction peaks

Figure 4 summarizes the XRD patterns of the CO_3_Ap HCBs, Cytrans Granules^®^ (CO_3_Ap granules), and HAp standard substance (STD). All samples demonstrated apatitic patterns characteristic of an apatite crystalline phase. The HAp STD showed sharp peaks that indicated high crystallinity, whereas the CO_3_Ap HCBs and Cytrans Granules^®^ showed broader peaks, suggesting low crystallinity.

**Fig. 5:**
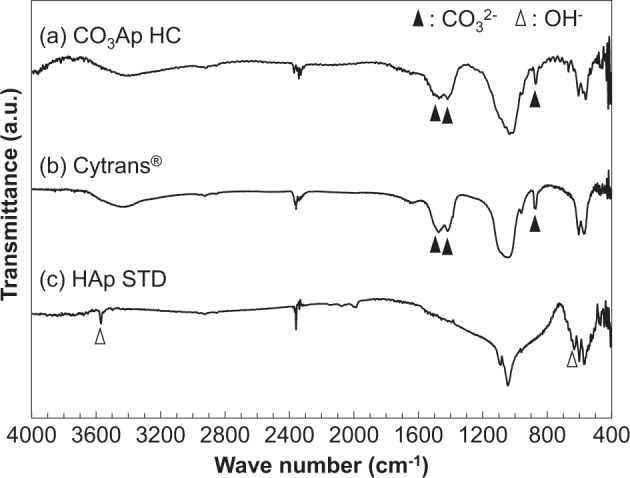
FT-IR spectra. CO_3_Ap HCBs (HC) (**a**), Cytrans Granules^®^ (Cytrans^®^; CO_3_Ap granules) (**b**), and HAp standard substance (STD) (**c**). Black triangles correspond to the B-type CO_3_^2−^ peaks (1455, 1410, and 875 cm^−1^)

The FT-IR spectra of the CO_3_Ap HCB, Cytrans Granules^®^, and HAp STD are shown in Fig. 5. Absorption attributed to CO_3_^2-^ (▲) in B-type CO_3_Ap was observed at 1455, 1440, and 875 cm^−1^ for the CO_3_Ap HCBs and Cytrans Granules^®^, whereas no such absorption was observed for the HAp STD. By contrast, absorption attributed to OH^-^ (△) was observed at 3568 and 627 cm^−1^ for the HAp STD, whereas no such absorption was observed for the CO_3_Ap HCBs. CO_3_Ap HCBs and Cytrans Granules^®^ showed almost the same XRD pattern and FT-IR spectra.

The carbonate content of the CO_3_Ap HCB was 11.1% ± 1.2%, almost identical to that of the Cytrans Granules^®^ (11.8 ± 0.6%) [7].

### Imaging analysis

**Fig. 6:**
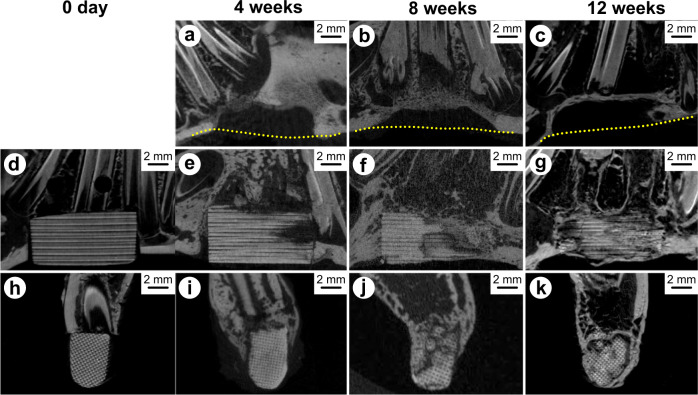
μ-CT images. The empty defect (**a**–**c**) and CO_3_Ap HCB (**d**–**k**) at day 0 (immediately after implantation) and 4, 8, and 12 weeks after implantation. The dotted lines (**a**–**c**) show the removed mandibular border. Sagittal sections (**a**–**g**) and cross-sections (**h**–**k**) of the pores

Figure 6 shows typical μ-CT images of an empty defect and a CO_3_Ap HCB at day 0 (immediately after implantation), and 4, 8, and 12 weeks after implantation. With no reconstruction (Fig. 6a–c), no continuity of the inferior border of the mandibular bone was observed during the observation period, indicating that this defect was a critical size defect and had cured with a hollow border. By contrast, when the defect was reconstructed with the CO_3_Ap HCB, the continuity of the inferior border of the mandibular bone was repaired, as assessed in the implantation samples (Fig. 6e–g). Modest resorption of the CO_3_Ap HCB was observed 4 weeks after implantation (Fig. 6e). At 8 and 12 weeks after implantation, the resorption of the CO_3_Ap HCB progressed with time, although the implanted HCB remained in place and retained the HC structure (Fig. 6f, g). The cross-sections obtained 4 and 8 weeks after implantation showed that the mineral bonelike structure contiguous to the bone defect end was visible at the outside of the HCB (Fig. 6i, j). At 12 weeks after implantation, the mineral bonelike structure completely surrounded the HCB (Fig. 6k).

### Histological analysis

**Fig. 7:**
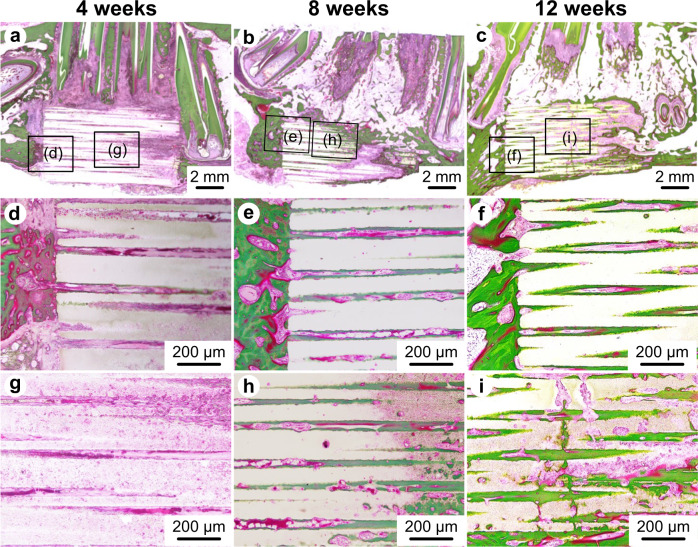
Typical histological images resulting from Villanueva Goldner staining of CO_3_Ap HCBs. At 4 (**a**, **d**, **g**), 8 (**b**, **e**, **h**), and 12 weeks (**c**, **f**, **i**) after implantation

Figure 7 shows typical histological images with Villanueva Goldner staining of the CO_3_Ap HCBs 4, 8, and 12 weeks after reconstruction. Few inflammatory cells or foreign body giant cells were observed regardless of the period after reconstruction. The resorption of CO_3_Ap HCBs was observed and radiographical evaluation was performed using the μ-CT images (Fig. 7a–c). Only modest resorption of CO_3_Ap HCBs occurred 4 weeks after reconstruction (Fig. 7a). At 8 and 12 weeks after reconstruction, the partial resorption of the CO_3_Ap HCBs progressed with time, although the HCBs remained in place and retained the HC structure (Fig. 7b, c). At 4 weeks after reconstruction, the bone extending from the existing bone was in contact with the lateral ends of the CO_3_Ap HCB, and a small amount of bone was observed in unidirectional continuous pores of the lateral parts at both ends but not in the central part of the CO_3_Ap HCB (Fig. 7a, d, g). At 8 and 12 weeks after reconstruction, calcified bone was observed in the pores of the lateral parts at both ends and in the central part of CO_3_Ap HCBs (Fig. 7b, c, e, f, h, and i). Furthermore, cross-linked structures of the bone between neighbor continuous pores were observed 12 weeks after reconstruction (Fig. 7i).

**Fig. 8:**
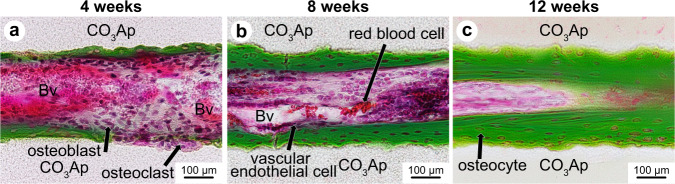
High-magnification images displaying the Villanueva Goldner staining of CO_3_Ap HCBs. At 4 (**a**), 8 (**b**), and 12 weeks (**c**) after implantation. Blood vessels (Bv) were found in the pores of CO_3_Ap HCBs. New bone was formed along the pores of the CO_3_Ap HCB and the thickness of the new bone increased over time

High-magnification images showed new bone formation along the continuous pores of CO_3_Ap HCBs (Fig. 8). Some osteoclasts invaded the wall of CO_3_Ap HCBs, forming resorption pits 4 weeks after implantation (Fig. 8a). In the neighboring environment, osteoblasts formed new bone (Fig. 8a). The thickness of which increased over time (Fig. 8b, c). Many osteoblasts were observed on the surface of the newly formed bone, and osteocytes were observed within the bone matrix. Numerous vascular endothelial cells and red blood cells were also found inside most of the pores, indicating the formation of blood vessels. Additionally, few inflammatory cells or foreign body giant cells were observed in the pores.

**Fig. 9:**
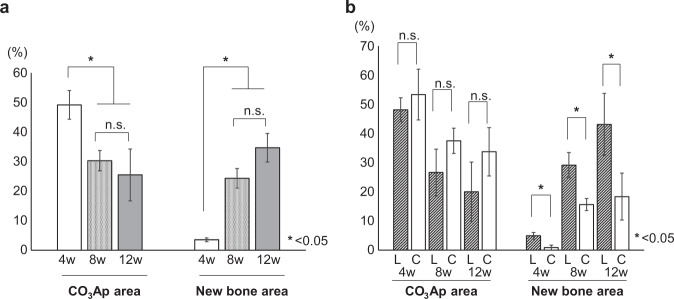
Comparison of new bone area and residual CO_3_Ap area. Percentage of new bone and residual CO_3_Ap 2, 4, and 8 weeks after implantation (**a**). *p < 0.05. Percentage of new bone and residual CO_3_Ap 2, 4, and 8 weeks after implantation in the central part (C) and in the lateral parts at both ends (L) (**b**). *p < 0.05

The percentages of the remaining CO_3_Ap area and calcified bone area (newly formed bone) stained green were estimated from the histological images (Fig. 9a). The CO_3_Ap area decreased over time. The calcified bone area stained in green increased with time. The following results at 4, 8, and 12 weeks after implantation were obtained (in%): CO_3_Ap area was 49.1 ± 4.9, 30.3 ± 3.5, and 25.5 ± 8.8, whereas calcified bone area stained in green was 3.0 ± 0.6, 24.3 ± 3.3, and 34.7 ± 4.8, respectively.

In addition, the percentages of the remaining CO_3_Ap area and calcified bone area stained green were estimated by dividing them into three parts: the lateral parts at both ends and the central part (Fig. 9b), and the following results at 4, 8, and 12 weeks after reconstruction (in%) were obtained: CO_3_Ap area in the lateral parts at both ends was 48.1 ± 4.1, 26.6 ± 8.0, and 20.0 ± 10.2, whereas CO_3_Ap area in the central part was 53.3 ± 8.7, 37.5 ± 4.3, and 33.7 ± 8.3; calcified bone area stained green in the lateral parts at both ends was 4.9 ± 1.1, 29.1 ± 4.3, and 43.1 ± 10.6; and calcified bone area stained green in the central part was 0.9 ± 0.9, 15.6 ± 2.1, and 18.3 ± 8.1, respectively.

## Discussion

Our results demonstrate that CO_3_Ap HCBs promote bone penetration along the unidirectional continuous pores from both lateral ends. The CO_3_Ap HCBs could restore the continuity of the bone in a 1 cm-long rabbit mandibular marginal bone defect. The CO_3_Ap HCBs showed excellent tissue response and good osteoconductivity and promoted the penetration of not only bone tissue but also blood vessels.

Ogose et al. reported that HAp is not resorbed and is entirely retained in human bone defects for periods longer than 130 months after implantation [18]. Furthermore, Hayashi et al. reported that HAp HCs are hardly resorbed at 12 weeks after implantation [19]. By contrast, beta-tricalcium phosphate (β-TCP) spontaneously dissolves under physiological conditions and in the absence of osteoclastic resorption [20, 21]. The β-TCP HCB is resorbed extremely rapidly and only a small amount remains 12 weeks after implantation [19].

Doi et al. reported that cultured osteoclasts resorb sintered CO_3_Ap disks [20]. CO_3_Ap is resorbed only via osteoclastic resorption, and its resorption rate and degree are similar to those of natural bone minerals [20–22]. Ishikawa et al. also reported that a CO_3_Ap HCBs are resorbed by osteoclasts and replaced with bone in rabbit femoral bone defects [15, 19]. The difference in resorption between CO_3_Ap and HAp was attributed to the presence of carbonate and the low crystallinity of CO_3_Ap. Spence et al. reported that a high carbonate content in carbonate-substituted hydroxyapatite (CHA) leads to increased osteoclastogenesis in culture and that the resorption of CHA increases with an increasing carbonate content [23]. Human bones contain 11% ± 1% of carbonate ions [24]. In the present study, the carbonate content of the CO_3_Ap HCB was set to be nearly equal to that of a human bone, and the results of CHN elemental analysis showed that the carbonate content of the CO_3_Ap HCB was 11.1% ± 1.2%.

The pores of biomaterials allow the passage of cells, delivery of oxygen and nutrients from the blood vessels, and removal of waste [25–27]. The pore size must be carefully controlled to improve the efficacy of bone regeneration [28, 29]. The optimum pore size for bone infiltration is debatable and depends on biomaterials such as titanium and calcium phosphate [13, 25, 30]. Frosch et al. and Taniguchi et al. reported that porous titanium implants with a pore size of approximately 600 μm are suitable for bone formation [31, 32].

HAp and β-TCP are representative calcium phosphates that are used as bone substitutes. Chang et al. reported bone ingrowth in porous HAp implants with different pore sizes (50, 100, 300, and 500 μm) in the proximal tibia of rabbits and concluded that the 300 μm pores in the HAp implants are of optimal size [33]. Kuboki et al. reported that the alkaline phosphatase activity and osteocalcin content are higher in bone morphogenetic protein-containing porous HAp blocks with a pore size of 300–400 μm than in those with different pore sizes (106–212, 212–300, 400–500, and 500–600 μm) when implanted subcutaneously in rats [34]. Moreover, infiltration of capillaries was observed in the porous HAp blocks with pore sizes greater than 300 μm. Taking these reports into consideration, a pore size ranging from 300 to 400 μm appears to be optimal for porous HAp.

Diao et al. studied biodegradable bone substrates, β-TCP scaffolds with a pore size of 100, 250, and 400 μm, in calvarial defects of rats at 4 weeks [35]. The use of porous β-TCP scaffolds with a pore size of 100 μm enabled a higher percentage of new bone ingrowth than the use of scaffolds with other pore sizes (250 and 400 μm); the porous β-TCP scaffolds with pores of both 250 and 400 μm were occupied by a larger amount of fibrous tissue [36]. A pore size of 100 μm appears to be optimal for porous β-TCP.

Akita et al. also reported a similar phenomenon in porous CO_3_Ap granules [14]. CO_3_Ap granules with a pore size of 120 μm showed the highest new bone formation inside the pores during the early stages among the various pore sizes (30, 50, 120, and 205 μm) [14]. Moreover, even though the pores sizes of 30, 50, and 120 μm were occupied by bone, fibrous tissue occupied the 205 μm pores of CO_3_Ap. Hence, it was suggested that the optimum pore size of CO_3_Ap is approximately 120 μm, which is smaller than that of HAp. By contrast, Hayashi et al. reported that bone formation in the pores of the CO_3_Ap HCB (macropores of 300 μm) is significantly superior to that in the 100 and 200 μm pores [36]. Thus, it was claimed that the effective pore size of the CO_3_Ap HCB for bone regeneration is between 200 and 300 μm, which is larger than that reported by Akita et al. [14]. The optimum pore size of CO_3_Ap for bone penetration remains controversial. The difference between the results of Akita et al. And Hayashi et al. could be attributed to the following three points: the CO_3_Ap HCB raw materials, the sectional shape of the pores, and the CO_3_Ap shape. The CO_3_Ap reported by Hayashi et al. was made from calcium hydroxide (Ca(OH)_2_), whereas that reported by Akita et al. and our team was made from CaSO_4_. In terms of sectional shape, square-shaped pores were found in the study by Hayashi et al. and in our study, whereas circular pores were found in the study by Akita et al. In the study by Hayashi et al. as well as in this study, the CO_3_Ap had a block shape (dimensions of approximately 5–10 mm); however, in the study by Akita et al, CO_3_Ap had a porous granule shape (dimensions of approximately 1–2 mm). More studies are needed to determine the optimal pore size of the CO_3_Ap HCBs.

In this study, CO_3_Ap area decreased with time: 49.1 ± 4.9, 30.3 ± 3.5, and 25.5 ± 8.8% at 4, 8, and 12 weeks after implantation, respectively. By contrast, calcified bone area stained in green increased: 3.0 ± 0.6, 24.3 ± 3.3, and 34.7 ± 4.8% at 4, 8, and 12 weeks after implantation, respectively. In short, CO_3_Ap HCBs were gradually resorbed and replaced by newly formed bone. New bone was formed even in the central part of the 1 cm CO_3_Ap HCB at 8 and 12 weeks after implantation. Moreover, in high-magnification images, some osteoclasts were observed to resorb the wall of CO_3_Ap HCBs, and osteoblasts also formed new bone in the neighboring environment at 4 weeks after implantation. This suggests that replacement of CO_3_Ap HCBs starts from osteoclast resorption in a manner similar to that of the bone remodeling.

Nagai et al. reported that cultured human bone marrow cells on CO_3_Ap disks promote osteoblastic differentiation earlier than when cultured on HAp disks, after 7 days of culture [2]. Ishikawa et al. reported that CO_3_Ap (Cytrans Granules^®^) histologically elicits a larger amount of new bone formation than HAp (Neobone^®^, CoorsTec, Tokyo, Japan) and β-TCP (Cerasorb^®^, Hakuho, Tokyo, Japan) in a hybrid dog mandible bone defect model 4 and 12 weeks after implantation [37]. Hayashi et al. reported that bone maturation is faster in the CO_3_Ap HCBs than in the HAp and β-TCP HCBs, and the amount of bone formation was higher in the CO_3_Ap HCBs than in the HAp and β-TCP HCBs in a rabbit rear limb defect model 4 and 12 weeks after implantation [19]. These results demonstrate that CO_3_Ap exhibits higher osteoconductivity than HAp and β-TCP.

Moreover, the structure of the HCBs has two merits for bone regeneration: 1) it can provide the space (continuous unidirectional pore) where new bone is formed and promote the infiltration of the bone from both lateral ends; 2) the wall of the pore can block the invasion of the soft tissue from the lateral sides. Consequently, more time is granted for bone infiltration and regeneration in the pores. Therefore, the CO_3_Ap HCB has an excellent ability to reconstruct relatively widespread bone defects caused by marginal mandibulectomy.

Substantial compressive strength that can withstand occlusal force is needed as a characteristic of reconstruction materials for mandibular bone defects. Misch et al. reported that the ultimate compressive strength of the trabecular bone in the human mandible ranges from 0.22 to 10.44 MPa, with a mean value of 3.9 MPa [38]. In the present study, the CO_3_Ap HCB was able to retain its form as a reconstructing material in the rabbit mandibular bone defect made via inferior border mandibulectomy during the observation period of 12 weeks. Regenos® (Kuraray Co., Tokyo, Japan) is a commercial HAp artificial bone with unidirectional macroporous structures similar to those of the CO_3_Ap HCB. The compressive strength of Regenos^®^ and the CO_3_Ap HCB is 14 MPa and 22.8 ± 3.5 MPa, respectively [19, 39]. In other words, the compressive strength of Regenos^®^ is considerably lower than that of the CO_3_Ap HCB. The straight struts present in the CO_3_Ap HCBs are separate, whereas the struts in Regenos^®^ are not completely straight and lean against each other. Orientation, linearity, and independence are critical in increasing the compressive strength along the macropores while maintaining a high porosity. Therefore, the CO_3_Ap HCBs structure seems to be an optimal structure because it possesses both high mechanical strength and high porosity.

Regenos^®^ is widely used in the orthopedic field in Japan for bone defects of the arm, leg, and vertebra, bearing large loads [40, 41]. It has already been applied in clinical cases to intra-articular calcaneal fractures with large bone defects and distal radius fractures and has yielded good results [42, 43]. The CO_3_Ap HCB has sufficient potential for clinical use in dental and orthopedic fields because it has higher compressive strength and osteoconductivity than Regenos^®^, which is composed of HAp. In the near future, we aim to further investigate the applicability of the CO_3_Ap HCBs in the treatment of larger bone defects, such as segmental mandibulectomy, with longer observation periods to assess its clinical use.

## Conclusions

This study is the first to report on the reconstruction of rabbit mandibular bone defects using the CO_3_Ap HCBs fabricated using the extrusion technique. The CO_3_Ap HCBs were gradually resorbed and replaced by newly formed bone. The morphology of CO_3_Ap HCBs not only promotes the bone infiltration from both lateral ends, but also inhibits the invasion of soft tissue from the lateral sides. The CO_3_Ap HCBs were found to have the capability to restore the continuity of 1-cm-long rabbit mandibular marginal bone defects. CO_3_Ap HCBs showed an excellent tissue response and good osteoconductivity and may be useful in the reconstruction of large mandibular bone defects.

## Supplementary information

Supplementary Figure
