# Supplementary figures and images for: Reconstruction of rabbit mandibular bone defects using carbonate apatite honeycomb blocks with an interconnected porous structure
Source: J Mater Sci Mater Med. 2022 Dec 31;34(1):2. doi: 10.1007/s10856-022-06710-2 (PMC9805415; doi:10.1007/s10856-022-06710-2)

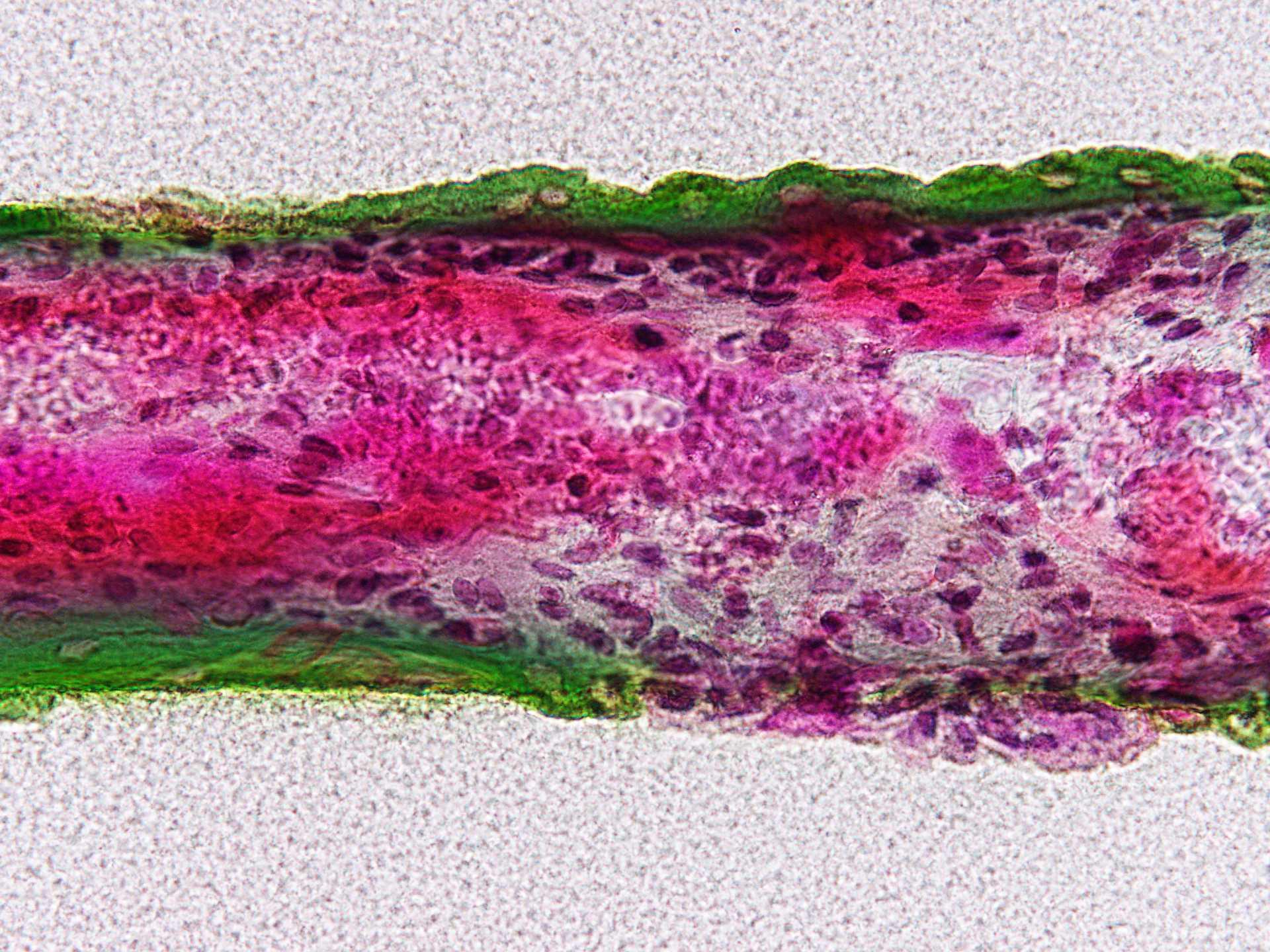

Supplement: Supplementary file 1 — Supplementary Figure [file 10856_2022_6710_MOESM1_ESM.tif]
